# Geographic variability of severe fever with thrombocytopenia syndrome virus genomes in the Miyazaki Prefecture, Japan: a cross-sectional molecular epidemiological study

**DOI:** 10.1186/s13071-026-07523-w

**Published:** 2026-06-30

**Authors:** Tsubasa Narita, Koji Yano, Miho Miura, Taro Nomachi, Maiko Shinden, Nami Tsuru, Sakura Onitsuka, Shuji Yoshino, Seigo Yamamoto, Shuji Ando, Tamaki Okabayashi

**Affiliations:** 1 Miyazaki Prefectural Institute for Public Health and Environment, Miyazaki-Shi, Miyazaki Japan; 2 National Institute of Infectious Diseases, Shinjuku-Ku, Tokyo Japan; 3 Department of Veterinary Science, Faculty of Agriculture, University of Miyazaki, Miyazaki-Shi, Miyazaki Japan; 4 Center for Animal Disease Control, University of Miyazaki, Miyazaki-Shi, Miyazaki Japan

**Keywords:** SFTS, SFTSV, Genotype, Whole genome sequence, Geographic distribution, Tick-borne disease, Zoonotic diseases, Japan

## Abstract

**Background:**

Severe fever with thrombocytopenia syndrome (SFTS) is a tick-borne disease caused by the SFTS virus (SFTSV). In Japan, the highest number of SFTS cases has been reported in the Miyazaki Prefecture. Despite previous genomic studies, the regional genomic diversity of SFTSV in Japan remains poorly characterized at the prefectural level. Hence, we aimed to analyze the whole genome sequencing of SFTSV strains collected in the Miyazaki Prefecture to assess their genomic diversity.

**Methods:**

Epidemiological data from patients were analyzed, and amplicon-based whole genome sequencing of SFTSV was performed using serum or plasma samples collected between 2012 and 2023 in the Prefecture. The resulting sequences were used to construct a phylogenetic tree, and the distribution of SFTSV genotypes was analyzed according to the presumed regions of infection.

**Results:**

The phylogenetic analysis revealed that four genotypes of SFTSV were present in the Prefecture, including currently unclassifiable genotypes and a novel subgenotype named J4. Analysis of the geographic distribution of each genotype revealed an association between genotypes and presumed regions of infection. Notably, potential genetic reassortment and recombination events were confirmed in Japanese strains.

**Conclusions:**

This study provides the largest whole genome sequencing of SFTSV conducted in Japan to date and confirms that SFTSV genotypes in the Miyazaki Prefecture exhibits strong regionality. Accordingly, we hypothesized that the dispersion of SFTSV through ticks and wild animals may be relatively limited. SFTSV genome analysis may help to estimate infection locations, particularly for cases with an unclear exposure history.

**Graphical Abstract:**

**Figure:**
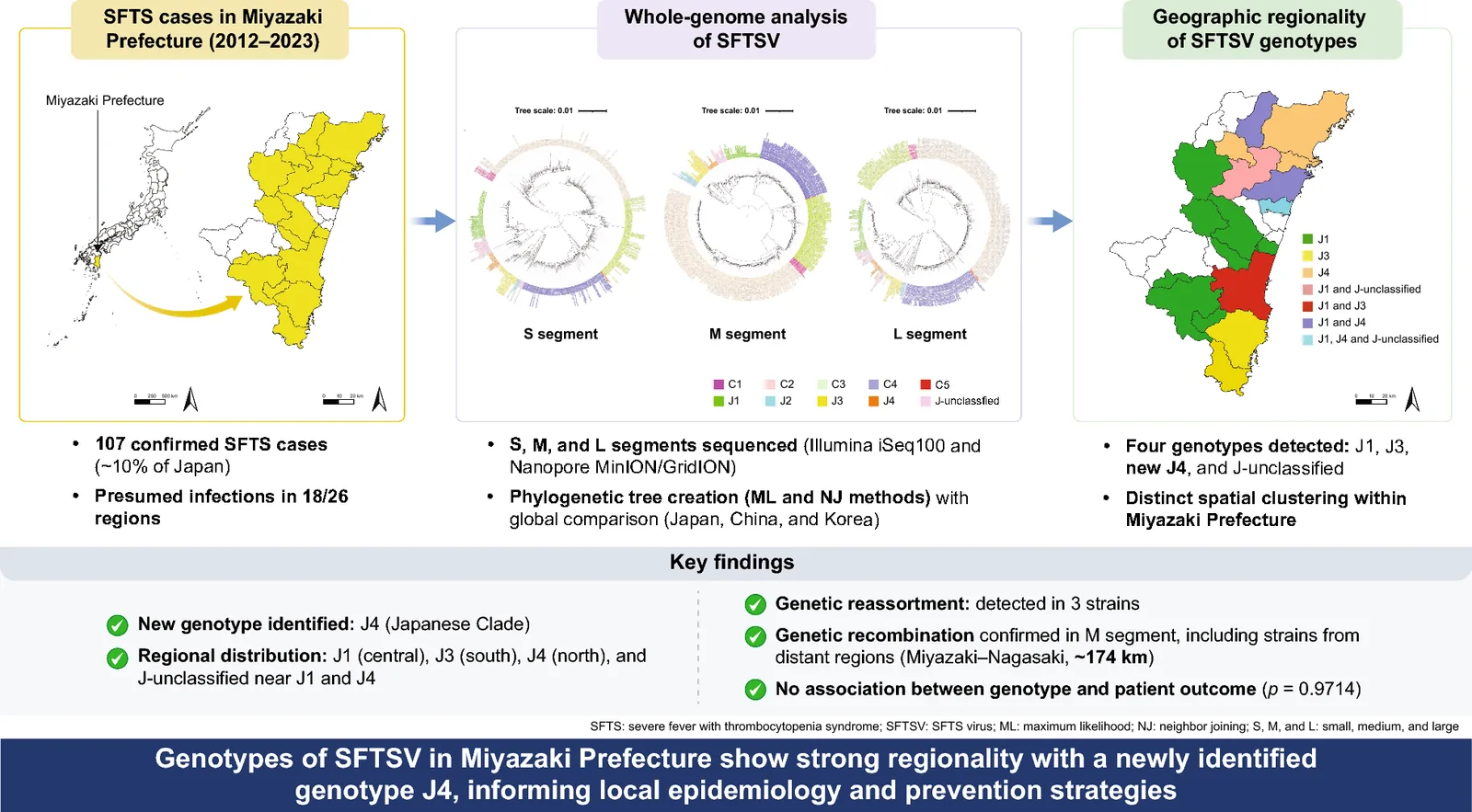

**Supplementary Information:**

The online version contains supplementary material available at https://doi.org/10.1186/s13071-026-07523-w.

## Background

Severe fever with thrombocytopenia syndrome (SFTS) is an infectious disease caused by the SFTS virus (SFTSV), which belongs to the genus *Bandavirus*, family *Phenuiviridae*, and order *Hareavirales*. SFTSV is a single-stranded negative RNA consisting of small (S), medium (M), and large (L) segments. This virus was discovered in China in 2011, and its presence in Japan was confirmed in 2013 [1, 2]. SFTS cases have been confirmed mainly in Asian countries, including China, Korea, Japan, Vietnam, Myanmar, Pakistan, Thailand, and Taiwan [3–8]. SFTS is a tick-borne infectious disease, and human infection is associated with bites from ticks such as *Haemaphysalis longicornis* or *Amblyomma testudinarium* [9, 10]. SFTSV genes and antibodies against SFTSV have been detected in animals other than humans, including dogs, cats, goats, sheep, cattle, pigs, chickens, geese, deer, shrews, minks, hedgehogs, swan geese, and spotted pigeons [11], making SFTS an important zoonotic disease. In Japan, a high seroprevalence of antibodies against SFTSV has been reported in deer, wild boars, and raccoons, suggesting that these animals may contribute to SFTSV transmission [12–14]. These findings suggest that SFTSV circulates between wild animals and ticks, with humans becoming incidentally infected. Previous reports have shown a correlation between forest perimeter and the number of SFTS cases, suggesting that human contact with the natural environment may be related to SFTS infection [15]. However, the precise transmission cycle of SFTSV in wild animal populations remains unclear, and several studies have suggested that birds may also be involved in its transmission [16–18]. It is certain that ticks play an important role in the spread of SFTSV.

In humans, the clinical manifestations of SFTS include fever, gastrointestinal symptoms, headache, muscle pain, neurological symptoms, swollen lymph nodes, subcutaneous bleeding, and bloody stool [19]. The fatality rate of patients with SFTS is approximately 6.3–30%, and is higher in older adults than in other age groups [20].

In Japan, the number of patients with SFTS has reached 930 since the first case of SFTS was reported in 2013, with 103 reported fatalities (as of October 31, 2023). The Miyazaki Prefecture, located in the south of Japan, has reported the highest number of patients with SFTS in the country. As of December 31, 2023, the cumulative number of patients in Miyazaki Prefecture was 107, with 30 deaths, accounting for approximately 10% of the cumulative number of patients in Japan; this suggests the potential for relevant analyses using a large number of clinical samples from this prefecture.

While previous studies in China and Korea performed whole genome sequencing of SFTSV in human patients [21, 22], few have been conducted in Japan [23, 24]. Furthermore, despite previous genomic studies, the regional genomic diversity of SFTSV at the prefecture level in Japan remains underexplored.

As SFTSV is a segmented RNA, reassortment can occur. However, it requires the coexistence of different genotypes. Li et al. [21] have suggested that ticks are crucial for the generation of such reassortants. Therefore, understanding regional genomic diversity is important for clarifying the role of ticks in the evolution of SFTSV.

Moon et al. [25] investigated the geographic distribution of M segments in the Gangwon Province, Korea; however, this previous study did not involve whole genome sequencing. In the present study, we aimed to analyze the regional genomic diversity of SFTSV, focusing on the Miyazaki Prefecture, based on the presumed region of infection of patients obtained from epidemiological surveys. We examined the regional genomic diversity of SFTS using whole genome sequencing.

## Methods

### Patients with SFTS

From April 2012 to December 2023, the Miyazaki Prefectural Institute for Public Health and Environment confirmed 111 SFTS cases. In this study, excluding 1 case identified at the University of Miyazaki, serum or plasma samples collected from 110 patients with SFTS were used for whole genome sequencing of SFTSV. Additional file 1 provides details on the number of cases at each stage of the study.

### Review of epidemiological information of patients

We analyzed the epidemiological information of the number of SFTS patients by year and month, number of fatal and non-fatal SFTS cases by age, number of fatal and non-fatal SFTS cases by sex, age in fatal and non-fatal SFTS cases, presumed cause of infection, animals that the patient had contact with, and symptoms experienced by the patient (excluding fever).

The association between fatality and sex was examined using *χ*^2^ tests, and the relationship between fatality and age was analyzed using the Wilcoxon rank sum test. Statistical analyses were performed using R (version 4-4-2; https://www.r-project.org). A significance level of *p* < 0.05 was used for all statistical analyses. As multiple statistical tests were performed in this study, *p*-values were adjusted using the Benjamini–Hochberg (BH) method.

### SFTSV gene detection by reverse transcription polymerase chain reaction (RT-PCR)

The presence of SFTSV genes in samples was confirmed using SFTSV-specific RT-PCR [26]. First, RNA was extracted from serum or plasma samples using the QIAamp viral RNA mini kit according to the manual (QIAgen, Hilden, Germany). PCR was performed targeting two sites in the NP region of the S segment using the primer sets listed in Table 1 and the SuperScriptIII One-Step RT-PCR with Platinum Taq (Thermo Fisher Scientific, Inc., Waltham, MA, USA) using the Thermal Cycler Gene Atlas G02 (Astec, Fukuoka, Japan) under the following conditions: 55 °C/30 min → 95 °C/2 min → (94 °C/30 s → 52 °C/30 s → 68 °C/30 s) 45 cycles → 68 °C/5 min → 4 °C/∞.

**Table 1:** RT-PCR primers for detecting SFTSV

		Sequence(5′→3′)	Target gene (position)
Primer set 1	Forward: SFTSV NP-1F	ATCGTCAAGGCATCAGGGAA	SFTS NP (202–221)
	Reverse: SFTSV NP-1Rd	TTCAGCCACTTCACCCGRA	SFTS NP (641–659)
Primer set 2	Forward: SFTSV NP-2F	CATCATTGTCTTTGCCCTGA	SFTS NP (168–187)
	Reverse: SFTSV NP-2R	AGAAGACAGAGTTCACAGCA	SFTS NP (609–628)

The amplified products were subjected to electrophoresis using a 1.4% agarose gel, and then photographed using ChemiDoc XRS + (Bio-Rad Laboratories, Inc., Richmond, CA, USA). Samples wherein a band appeared at the same position as that of the positive control (approximately 400 bp) were considered positive samples. If only one set of primers was positive, the sample was considered positive [26].

The positive control pCR-SFTSV-posicon constructed by Yoshikawa et al. [27] was kindly provided by the National Institute of Infectious Diseases. This diagnostic method was performed in accordance with the SFTS diagnostic manual established by the National Institute of Infectious Diseases.

### Whole genome sequencing

We performed whole genome sequencing on 98 S, 102 M, and 104 L segment samples of 110 SFTSV-positive serum or plasma samples collected from 2012 to 2023, excluding those previously analyzed by Yoshikawa et al. [23]. Whole genome sequencing was performed based on the amplicon sequencing method by Quick et al. [28] for Zika virus and the ARTIC method used in the whole genome sequencing of SARS-CoV-2 (https://www.protocols.io/view/ncov-2019-sequencing-protocol-bbmuik6w). Primers were prepared using the Primal scheme (version 1.3.2; https://primalscheme.com/). Whole genome sequencing was performed separately for each segments using an Illumina iSeq100 (Illumina, San Diego, CA, USA), Nanopore MinION Mk1C (Oxford Nanopore Technologies, Oxford, UK), and Nanopore GridION (Oxford Nanopore Technologies, Oxford, UK). Primers and protocols for sequencing using the Illumina iSeq100 (https://www.protocols.io/view/severe-fever-with-thrombocytopenia-syndrome-virus-3byl41mdolo5/v2), Nanopore MinION, and GridION (https://www.protocols.io/view/severe-fever-with-thrombocytopenia-syndrome-virus-261ge8qy7g47/v2) are provided in protocols.io.

### Sequencing and construction of phylogenetic trees

Nucleotide sequences were obtained using Galaxy (Galaxy (http://usegalaxy.org), Galaxy Europe (https://usegalaxy.eu), and NanoGalaxy (https://nanopore.usegalaxy.eu).

**Fig. 1:**
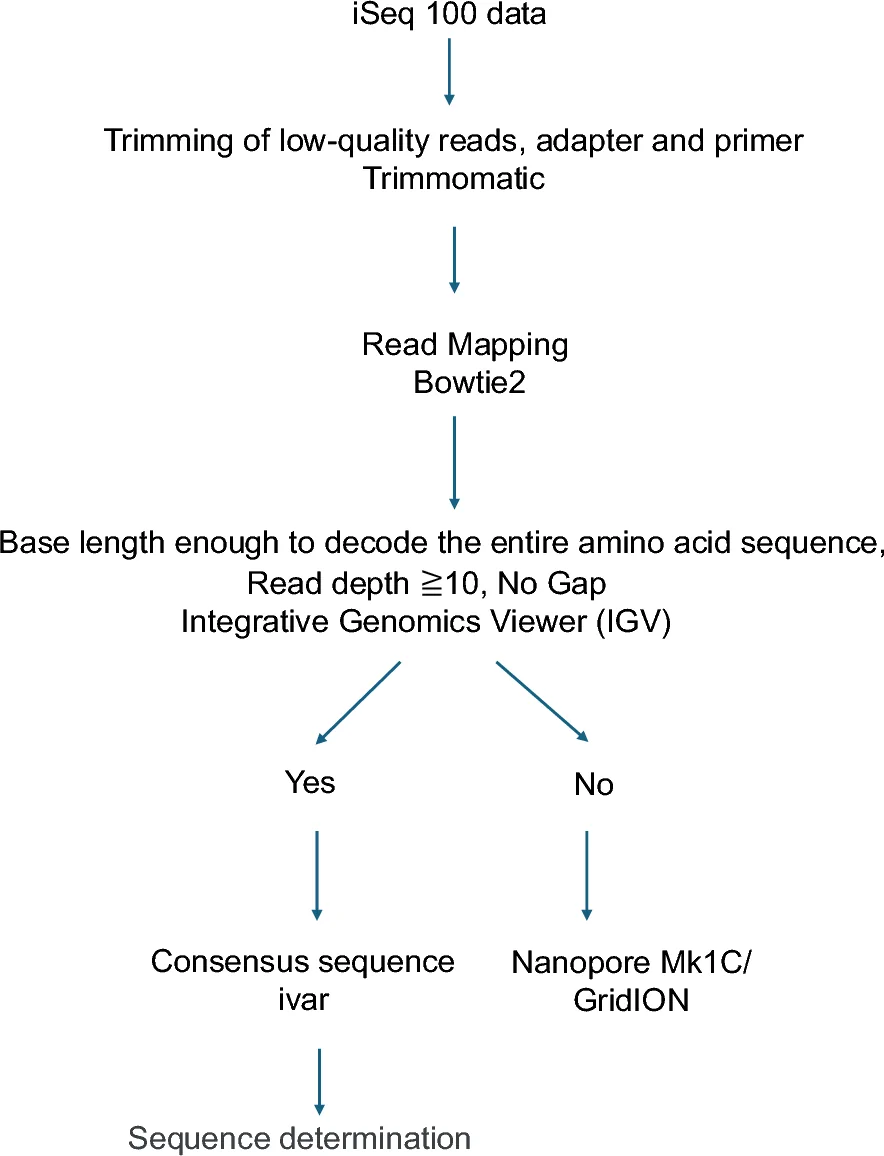
Data analysis flow (iSeq100). Process for obtaining sequences from data analyzed by iSeq100. Galaxy (http://usegalaxy.org) and Galaxy Europe (https://usegalaxy.eu) were used for analysis. Trimmomatic [29] was used for trimming of low-quality reads, adapters, and primers, Bowtie2 [30, 31] for read mapping, IGV tool [32–35] for visualization of sequences, read depth, and read coverage, and ivar [36] for determination of consensus sequence

**Fig. 2:**
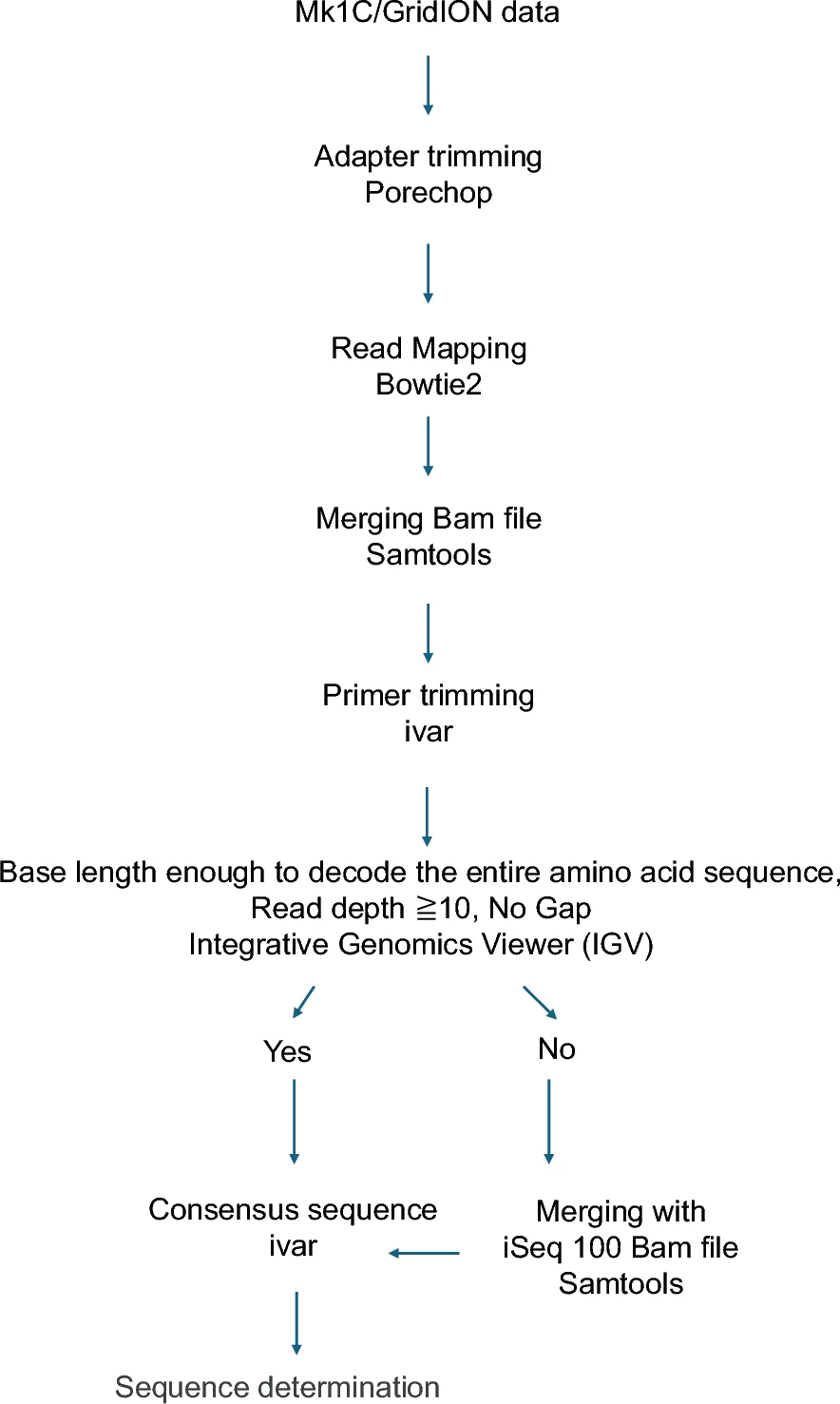
Data analysis flow (MinION Mk1C/GridION). Process of obtaining sequences from data analyzed by MinION Mk1C or GridION. NanoGalaxy (https://nanopore.usegalaxy.eu) was used for the analysis. Porechop [37] was used for adapter trimming, Bowtie2 [30, 31] for read mapping, Samtools [38] for merging Bam file, ivar [36] for primer trimming and determination of consensus sequence, and IGV tool [32–35] for visualization of sequences, read depth, and read coverage

Only sequences that met all of the following criteria were included in the analysis: sufficient base length to cover the entire amino acid sequence, read depths ≥ 10, and absence of gaps. The detailed process for obtaining nucleotide sequences is shown in Figs. 1 and 2 [29–38].

To make regionality easier to understand, three strain groups were constructed using MAFFT (version 7.526; https://mafft.cbrc.jp) [39, 40]: the Miyazaki strain group, comprised by collected strains from the Miyazaki Prefecture; the Japanese strain group, comprised by strains from Japan; and the Global strain group, comprised by strains from Japan, Korea, and China.

The phylogenetic tree of the Global strain group (Global tree) was constructed using the neighbor joining (NJ) (MEGA11 [version 11.0.8; https://www.megasoftware.net]) [41] and maximum likelihood (ML) methods (IQ-Tree [version 2.4.0; http://iqtree.cibiv.univie.ac.at]). Phylogenetic trees of the Japanese (Japanese tree) and Miyazaki strain groups (Miyazaki tree) were constructed using the NJ method. The IQ-Tree analysis selected the best model based on Akaike's Information Criterion (AIC). The reference sequence of SFTSV was constructed on the basis of data published previously [23, 42]. The obtained nucleotide and reference sequences are shown in Additional file 2. The reference sequences were only those obtained from human samples wherein the genomes had been almost completely sequenced.

To the best of our knowledge, no method has been established for the phylogenetic classification of SFTSV. In Japan, the J1–J3 and C1–C5 classifications are used, while in other countries, the A–F classification is used. In this study, we used the Japanese method for classification because the A–F classification method classifies Japanese strains into almost one genotype, making it difficult to grasp regional differences in genotypes within Japan [21–24].

New genotypes were determined by considering phylogenetic tree structure, node support values, and pairwise distances. The new genotype criteria for the phylogenetic tree structure were based on the requirement that the genotype be maintained in all segments within all three strain groups. The new genotype criteria for node support values were defined as a bootstrap value of 80% or higher in the ML phylogenetic tree for all segments. As the new genotypes are a reclassification from J1, which was previously classified by Yoshikawa et al. [23], we confirmed the pairwise distances between J1 and the new genotypes. Recombinant strains were excluded from the calculation of gene distance. The new genotype criteria for phylogenetic tree structure required that the genotype be consistently maintained across all segments and strain groups, with bootstrap values ≥ 80% in ML trees. As no established standard exists for pairwise distances in SFTSV, a threshold of less than 2% within genotypes and ≥2% between genotypes was applied as a supplementary criterion based on previous reports [43, 44], rather than as a strict cutoff. The pairwise distances (between and within genotypes) were calculated using MEGA11 (Kimura 2-parameter model [bootstrap:1000]). Based on the classified genotypes, the relationship between genotypes and patient outcomes was analyzed using the *χ*^2^ test in R (version 4-4-2; https://www.r-project.org). The effect size was assessed using Cramér’s V.

### Regionality of SFTSV genotypes

The regionality of genotypes in the Miyazaki Prefecture was investigated using phylogenetic trees of SFTSV and information on presumed regions of infection of patients. The regionality of genotypes was classified according to the branching of phylogenetic trees. Subsequently, haplotype networks of the Miyazaki strain group were created using PopART (version 1.7; https://popart.maths.otago.ac.nz), and the regionality of SFTSV genotypes in the Miyazaki Prefecture was reanalyzed [45, 46]. The regional characteristics of genotypes were shown on a map of the Miyazaki Prefecture. Additionally, by comparing the Japanese, Global, and Miyazaki trees, we confirmed the differences between Miyazaki and other regions.

### Genetic reassortment and recombination

According to the method of Li et al. [21], strains with different genotypes between segments were defined as strains wherein genetic reassortment occurred, and the reassortment was analyzed for the Miyazaki strain group. Reassortments that included J-unclassified strains were not considered.

Using a dataset from the Global strain group, the Miyazaki strain group was confirmed for genetic recombination using RDP4 (version 4.101; http://web.cbio.uct.ac.za) [47]. The recombination signals were confirmed for the full-length sequence of each segment according to the method of Park et al. [48], using GENECONV, BOOTSCAN, MaxChi, Chimaera, SiScan, 3Seq, and RDP. Strains that were confirmed to have the recombination signals (*p* < 0.05) by three or more methods were considered to be recombined [49–55], and manual verification of recombinant organisms was performed.

## Results

### Review of epidemiological information of patients

**Fig. 3:**
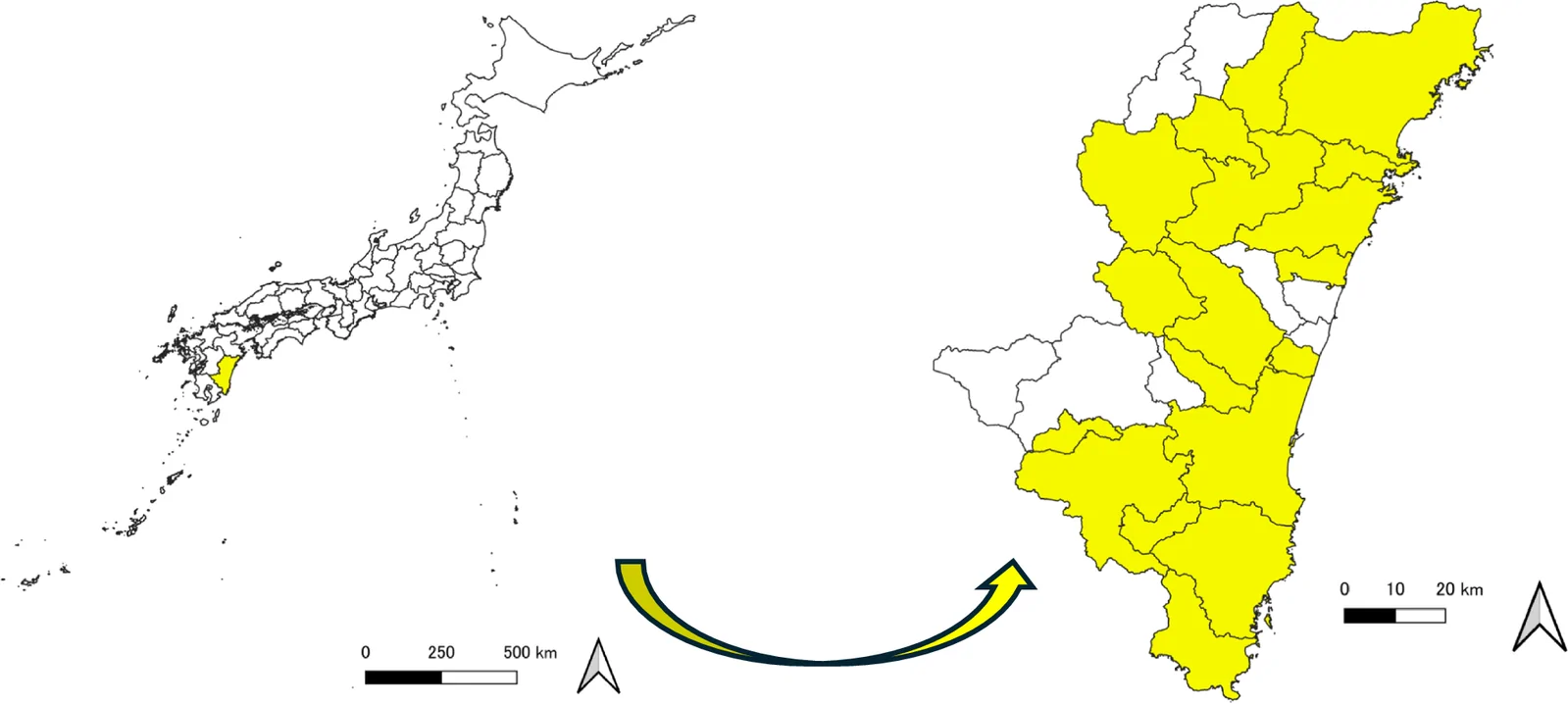
Presumed regions of infection of patients with severe fever with thrombocytopenia syndrome (SFTS) in the Miyazaki Prefecture. The yellow area on the map on the left shows the location of the Miyazaki Prefecture. The yellow areas on the map on the right indicate the presumed regions of infection of patients with SFTS from April, 2012 to December, 2023. These maps were drawn with QGIS (3.40.5-Bratislava, https://qgis.org) using data from the Geospatial Information Authority of Japan

**Fig. 4:**
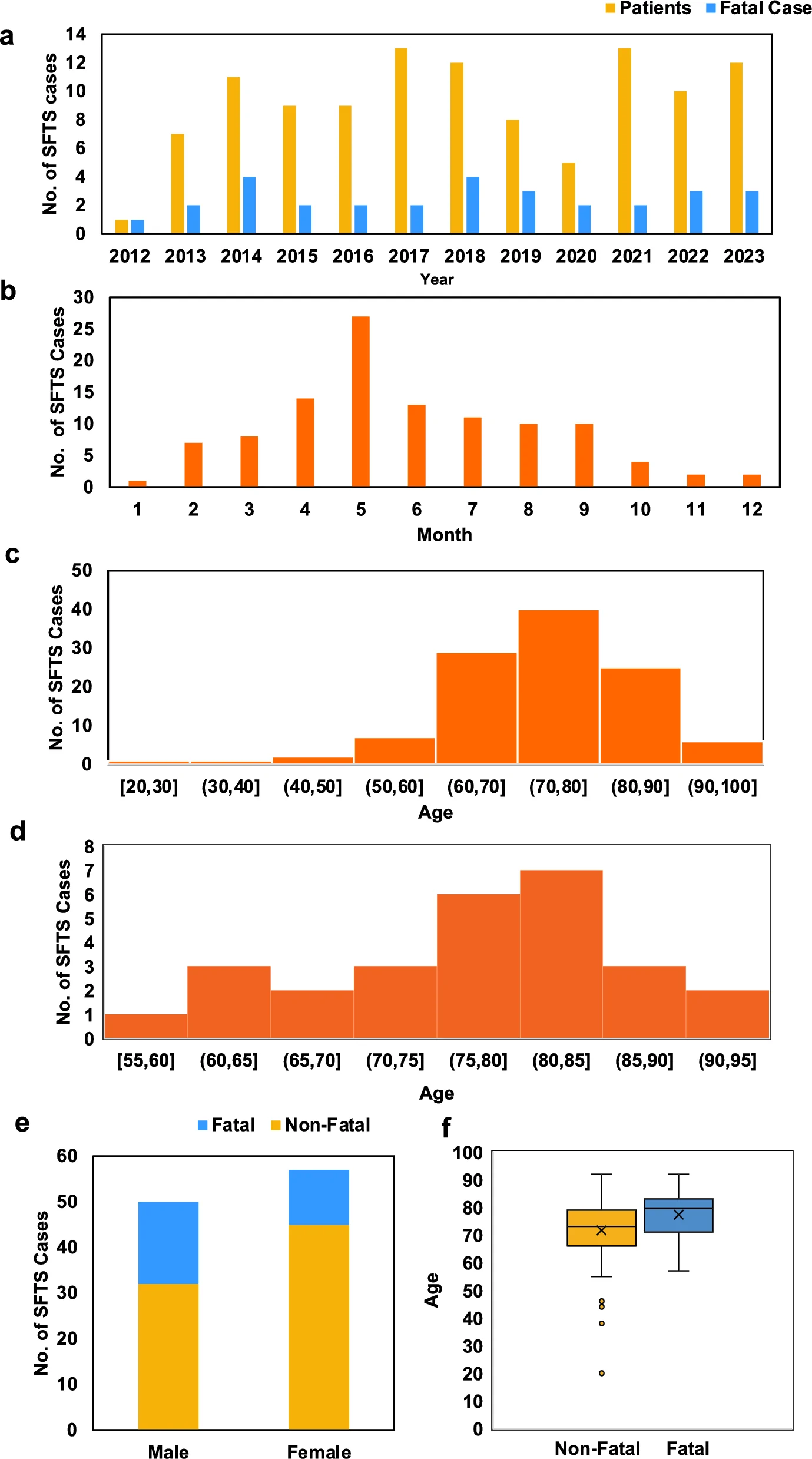
Epidemiology of patients with severe fever with thrombocytopenia syndrome (SFTS) in the Miyazaki Prefecture. Summary of the epidemiological information of patients with SFTS in the Miyazaki Prefecture. **a** Number of SFTS cases by year. **b** Number of SFTS cases by month. **c** Number of SFTS cases by age. **d** Number of fatal SFTS cases by age. **e** Number of fatal and non-fatal SFTS cases by sex. **f** Age of patients in fatal and non-fatal SFTS cases (*: *p* < 0.05, × : average)

**Fig. 5:**
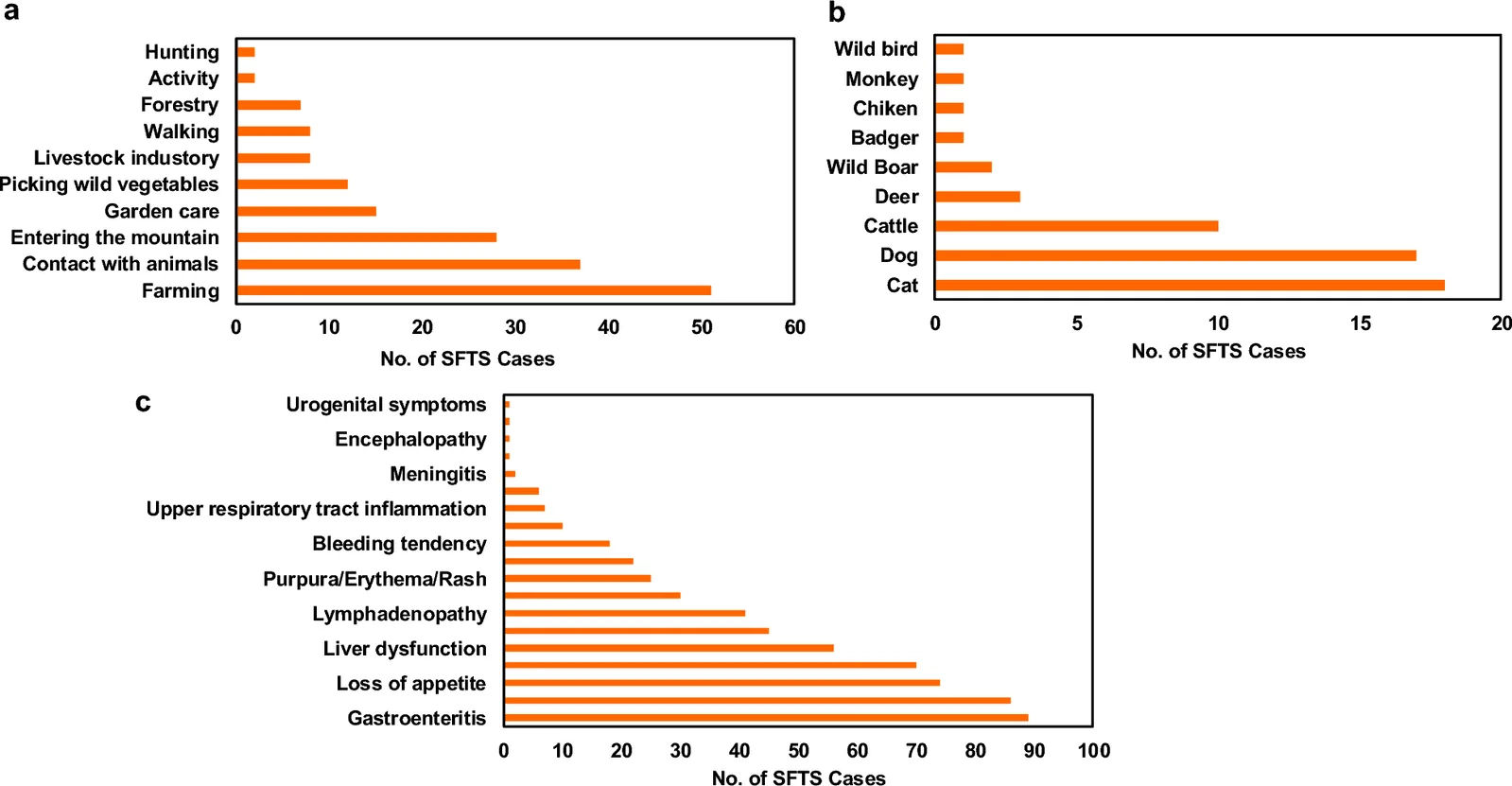
Infectious causes and symptoms in patients with severe fever with thrombocytopenia syndrome (SFTS). **a** Presumed cause of infection. **b** Animals with which the patient had contact. **c** Symptoms of the patient (excluding fever). Note that the total number of infectious causes was not equal to the number of patients, as some patients may have had multiple infectious causes

Epidemiological data on the 107 infection cases in the Miyazaki Prefecture, excluding the 4 cases from the other prefectures, are shown in Figs. 3, 4 and 5. These patients with SFTS were distributed evenly from the north to the south of Prefecture. Infection was identified in 18 (69.2%) of the 26 regions (cities and towns) (Fig. 3). The number of patients remained at approximately 10 cases per year (Fig. 4a), with most cases occurring in May (Fig. 4b).

The median age of patients (*n* = 111) was 75.0 years (interquartile range [IQR] 67–81; men: 72.5 years [IQR 65.75–80.25; *n* = 52]; women: 76.0 years [IQR 69.5–82.0; *n* = 59]). Most of the patients were 70–79 years of age (Fig. 4c). The median age of those who died was 79.5 years (IQR 71.75–82.75; men: 80.5 years [IQR 71.75–82.75], women: 78.5 years [IQR 74.75–81.25]).

Most of the patients who died were in their late 70s or early 80s (Fig. 4d). Of the 107 infected patients (50 men and 57 women), 30 died (18 men and 12 women; Fig. 4e), indicating a 28.3% fatality rate. No significant difference was observed between men and women in terms of fatality due to SFTSV infection (*χ*^2^[1] = 2.2552, *p* = 0.1332, BH: *p* = 0.19980). The Wilcoxon rank sum test showed no significant difference between the ages of fatal and non-fatal cases (*W* = 813.5, *p* = 0.01796, BH: *p* = 0.05388; Fig. 4f). The median time from onset of SFTS to hospital admission was 5 days (IQR 3–7) for fatal and non-fatal cases.

The most common patient activities before infection (including duplicate counts) included farming, having contact with animals, and entering the mountain (Fig. 5a). However, these counts are the number of patient activities before infection. A high count does not necessarily indicate the cause of infection (e.g., people with companion animals would have listed animals as the cause of contact; thus, the number of animal contacts would inevitably be high). The animals which the patients had contact with before infection (including duplicate counts) were primarily dogs and cats, followed by cows, wild boars, and deer (Fig. 5b).

Common patient symptoms included fever, gastroenteritis, fatigue, loss of appetite, liver dysfunction, neurological symptoms (including impaired consciousness), and lymph node swelling (Fig. 5c).

### Phylogenetic analysis

**Fig. 6:**
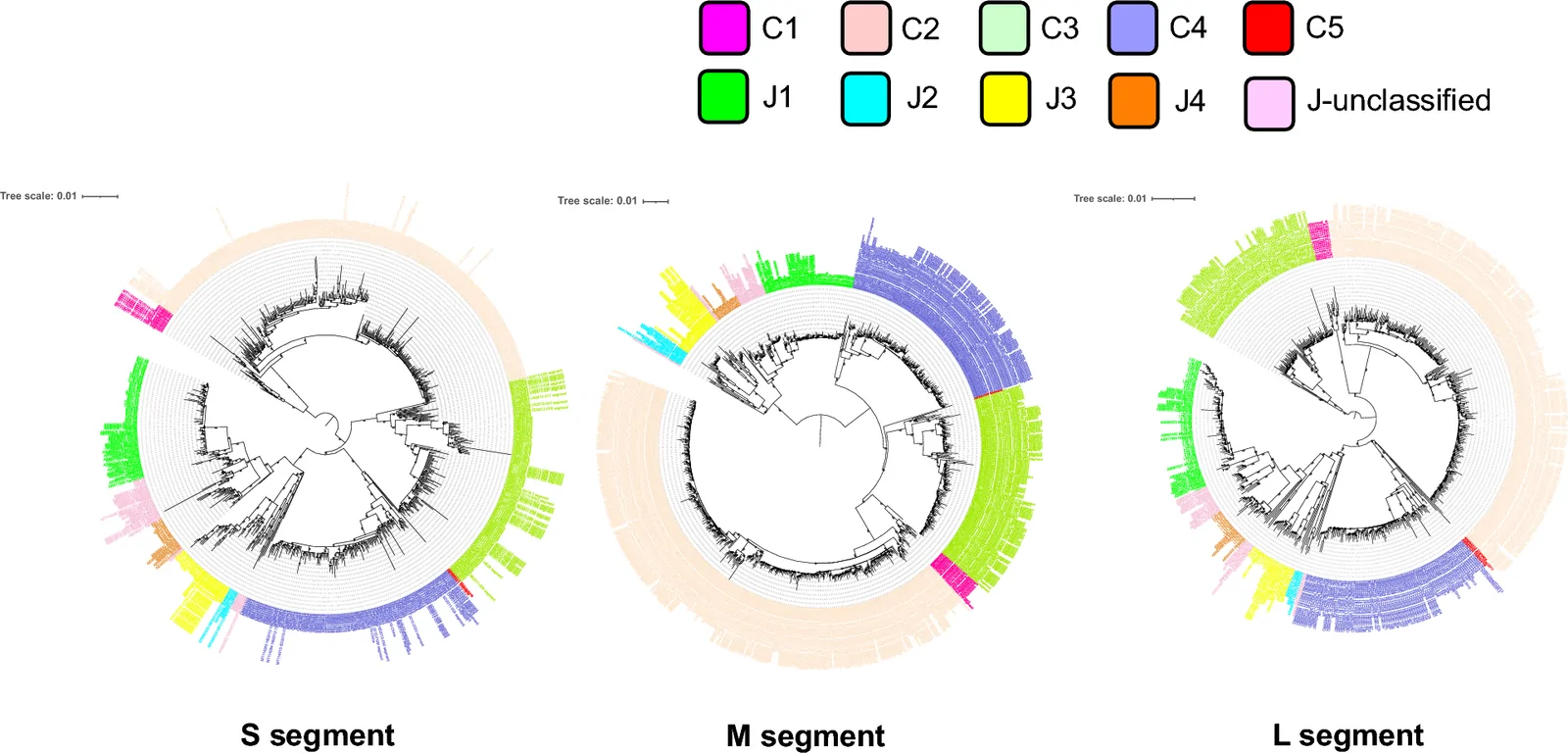
Global tree of S, M, and L segments by the maximum likelihood method. Phylogenetic trees drawn using 742, 710, and 547 samples from the S, M, and L segments, respectively, including the sequences obtained in this study. The phylogenetic trees were constructed using the ML method (bootstrap 1000 times) with IQ-Tree (version 2.4.0; http://iqtree.cibiv.univie.ac.at). iTOL (version 7; https://itol.embl.de) was used for phylogenetic tree visualization. See Additional file 2 for genotypes details

**Fig. 7:**
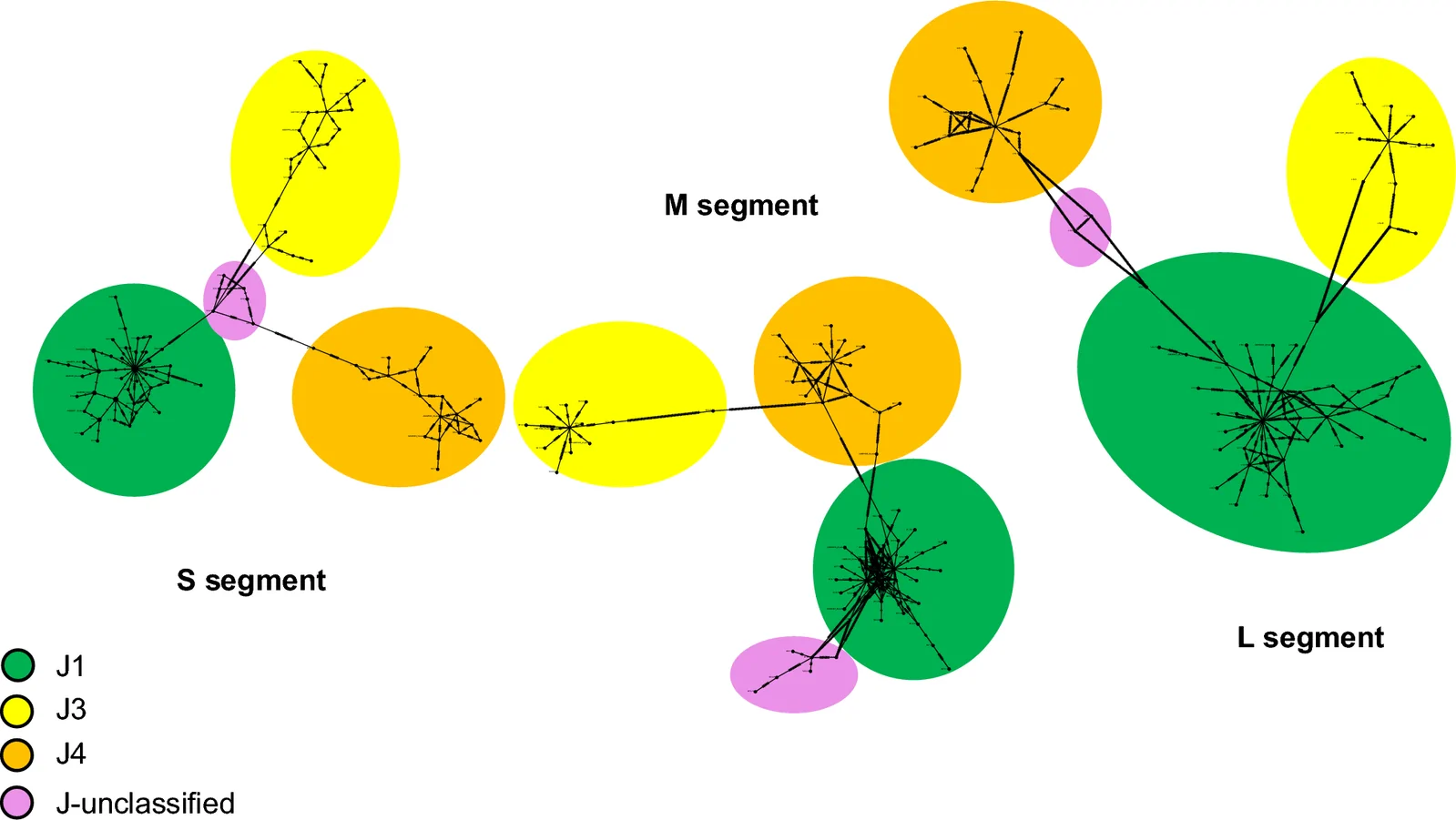
Haplotype network constructed using severe fever with thrombocytopenia syndrome virus (SFTSV) strains from the Miyazaki Prefecture, Japan. Haplotype network of SFTSV strains in the Miyazaki Prefecture, drawn using PopART, showing that the genotypes of SFTSV in the Miyazaki Prefecture could be classified into four types

In this study, the SFTSV whole genome sequences of 85 S, 72 M, and 76 L segment samples were newly determined (Additional file 2). The results of the phylogenetic tree analysis are shown in Fig. 6 and Additional file 3. Differences in recovery rates were observed among the genome segments, which may be attributed to variations in segment length, viral copy, and the number of samples processed per sequencing run. The best-fit models for the ML method used in the phylogenetic analyses were TVMe + R4 (AIC 59276.917) for the S segment, GTR + F + I + R5 (AIC 101271.034) for the M segment, and GTR + F + I + R5 (AIC 144556.233) for the L segment. The genotypes of SFTSV detected from patients in the Miyazaki Prefecture were classified into four groups: J1 and J3 (as described by Yoshikawa et al. [23]), a newly identified subgenotype J4, and a J-unclassified group. These J4 and J-unclassified genotypes are part of the genotypes classified as J1 in the study by Yoshikawa et al. [23]. A comparison with the Global tree (742, 710, and 547 of S, M, and L segments, respectively) confirmed that J4 belonged to the Japanese clade, but formed groups different from those of J1 and J3 (Fig. 6). J4 was also confirmed as a new subgenotype in the Miyazaki (97, 80, and 82 in the S, M, and L segments, respectively) and Japanese trees (148, 114, and 120 in the S, M, and L segments, respectively) and haplotype network analyses (Figs. 6 and 7; Additional file 3). Bootstrap values for Japanese clade were consistently > 90% for all segments (Fig. 6, Additional file 4). The pairwise distances between J1 and J4 in each segment are as follows: S segment (J1:0.01 ± 0.00, J4:0.01 ± 0.00, J1–J4:0.0197 ± 0.0029), M segment (J1:0.00 ± 0.00, J4:0.01 ± 0.00, J1–J4:0.0190 ± 0.0020), and L segment (J1:0.01 ± 0.00, J4:0.00 ± 0.00, J1–J4:0.0205 ± 0.0016). Although the pairwise distances in the S segment and M segment were slightly below the predefined threshold, the difference was marginal and all other criteria, including strong phylogenetic support and consistency across segments, were satisfied.

In the Japanese clade, most of the strains not included in J1, J3, and J4 were from prefectures other than Miyazaki. As future investigations may result in branching, they were classified as J-unclassified. However, J-unclassified is an unclassified, but not necessarily single, group. The J-unclassified group was confirmed to have formed at least two clusters (Putative J-1 and J-2) in the ML phylogenetic tree, which may represent new genotype candidates (Additional file 4).

Yoshikawa et al. [23] reported that the C5 genotype is present only in the M and L segments, and that the S segment is classified as belonging to the C4 genotype. However, the ML method in the present study confirmed the presence of C5 in the S segment (Fig. 6). In the Japanese clade, no differences were observed in the classification between the NJ and ML methods, except for that with C5.

**Fig. 8:**
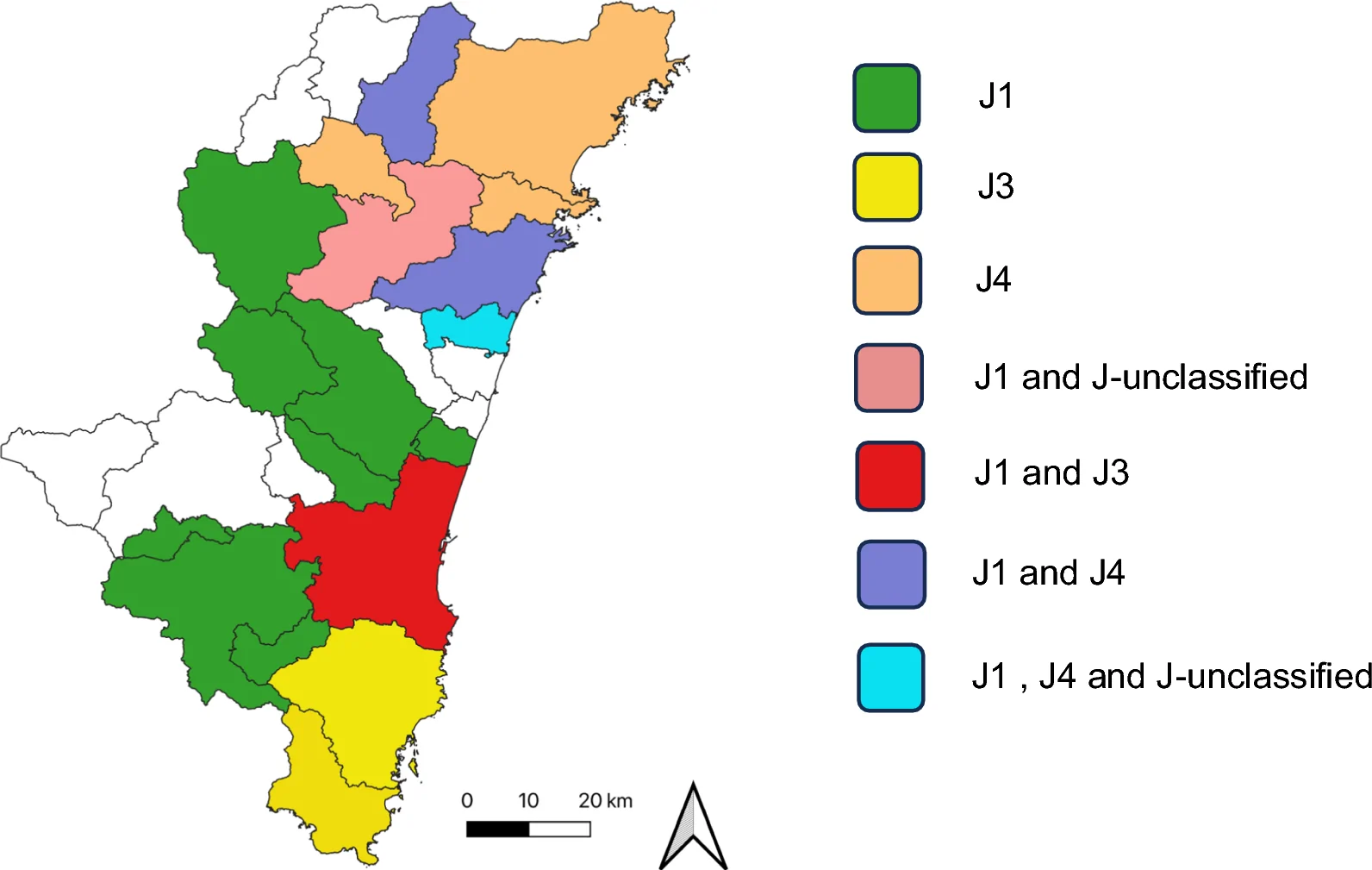
Association between genotypes and presumed regions of infection of patients with severe fever with thrombocytopenia syndrome (SFTS). Presumed regions of infection of patients for each of the four genotypes present in the Miyazaki Prefecture. This map was drawn with QGIS (3.40.5-Bratislava, https://qgis.org) using data from the Geospatial Information Authority of Japan. Each genotype was distributed in a close range

The distribution of genotypes in Miyazaki strains is shown in Fig. 8. J1, J3, and J4 were distributed in central, southern, and northern regions of the Miyazaki Prefecture, respectively. J-unclassified was confirmed near regions where J1 and J4 were also confirmed.

The four SFTSV strains from patients suspected to have been infected in two other prefectures belonged to the Japanese clade. The three strains from one of these two prefectures belonged to a J-unclassified genotype. Evaluation of the Japanese tree showed that some strains from multiple prefectures were mixed in each branch, except for those of J3 (Additional files 2 and 3). The Chinese and Korean strains included in J3 clearly branched out from the Japanese J3 strains in the Global tree (Additional file 3). No significant difference was observed between genotypes and patient outcome (*χ*^2^[2] = 0.058031, *p* = 0.9714, BH: *p* = 0.97140) (Table 2). The effect size was small (Cramér’s V = 0.02512), suggesting a weak association. Table 2 shows patient outcomes by genotype. For strains that were genotyped in any segment, the strains were genotyped accordingly. Table 2 does not include strains from other prefectures, strains classified as J-unclassified, or reassortment strains.

**Table 2:** Associations between genotypes and patient outcomes

	J1	J3	J4	Sum
Fatal	15	6	6	27
Non-fatal	37	13	15	65
Sum	52	19	21	92

### Occurrence of genetic reassortment and recombination in SFTSV

Potential reassortment was confirmed and identified in three strains (Table 3). The use of RDP4 revealed potential recombination signal detection in only the M segment (Table 4). A potential recombination signal in AB985298 was detected using four methods: MaxChi (*p* = 3.310e-3), Chimera (*p* = 3.193e-3), SiScan (*p* = 1.104e-2), and 3Seq (*p* = 6.247e-3), whereas potential recombination signal in LC876440 was detected using three methods: MaxChi (*p* = 6.589e-4), Chimera (*p* = 9.084e-5), and SiScan (*p* = 8.351e-6).

**Table 3:** Reassortment in SFTSV strains from the Miyazaki Prefecture

Strain	Segment L		Segment M		Segment S	
	Accession no.	Genotype	Accession no.	Genotype	Accession no.	Genotype
19–20	LC876363	J4			LC876450	J3
19–29	LC876340	J1	LC876369	J4	LC876490	J4
21–45	LC876314	J3			LC876516	J1

**Table 4:** Genetic recombination identified in SFTSV strains from the Miyazaki Prefecture

Segment (breakpoint)	Recombination			Major			Minor		
	Accession no.	Genotype	Prefecture	Accession no.	Genotype	Prefecture	Accession no.	Genotype	Prefecture
M 658(512–858)	AB985298	J4	Miyazaki	LC876382	J4	Miyazaki	AB817993	J1	Nagasaki
M 670(452–927), 1852(1750–1905)	LC876440	J3	Miyazaki	LC876436	J3	Miyazaki	LC876377	J4	Miyazaki

The results of RDP4 and manual verification are shown in Additional File 5. All the recombination occurred between strains detected in geographically distant locations, however one strain in particular was a recombination with a strain from outside the Miyazaki Prefecture (i.e., in the Nagasaki Prefecture). Table 3 shows the strains for which sequences were determined in this study, wherein potential reassortment was confirmed. The reassortment including J-unclassified strains was excluded. The straight-line distance between the Miyazaki and Nagasaki prefectures is approximately 174 km.

Table 4 lists potential genetic recombination of SFTSV strains from the Miyazaki Prefecture confirmed in this study. Potential recombination strain resulted from major and minor strains. The number in parentheses next to “Breakpoint” represents the 99% confidence interval.

## Discussion

In this study, we performed whole genome sequencing of SFTSV, defined a new subgenotype (i.e., J4), and revealed the regional distribution of SFTSV genotypes in the Miyazaki Prefecture. Additionally, to the best of our knowledge, we detected potential genetic reassortment and recombination signal for the first time in Japanese SFTSV strains.

Epidemiological surveys showed that many of the patients infected with SFTSV in the Miyazaki Prefecture were older adults (Fig. 4c). The underlying reasons remain unclear; however, we hypothesize that their immune status may be associated with disease severity. Kim et al. [56] showed that age-associated immune dysregulation is related to the severity of SFTS in ferrets. In this study, although the number of deaths was high among men, the total number of cases was higher among women. However, no significant association was observed between sex or age and fatality rate, suggesting that infection control measures are necessary regardless of age or sex. All patient infections were associated with outdoor activities, suggesting that tick prevention measures in outdoor settings are crucial for preventing infections. The list of infectious causes included items such as “Entering the mountain”, “Picking wild vegetables”, and “Forestry” suggesting that human contact with natural environments increases the risk of SFTS infection. Symptoms of patients included gastroenteritis, fatigue, and loss of appetite, making it difficult to distinguish SFTS from other illnesses based on symptoms alone.

Previous studies of whole genome sequencing of SFTSV [21–24] performed sequencing after cell culture. However, this method detects only virus strains with cell-adapted mutations [57, 58]. Conversely, in the present study, whole genome sequencing was performed directly on patient serum or plasma samples. Generally, obtaining results from samples with low viral loads is difficult when sequencing is performed directly on clinical samples. Nonetheless, herein, sequencing samples with low viral loads was possible to a certain extent by amplifying viral genes using multiplex PCR. Sequencing directly from clinical samples has the advantage of providing faster results than cell cultures. Furthermore, we consider it more reasonable, as it eliminates the possibility of viral mutations during cell culture and allows for identification of diverse viral genotypes, including quasi-species, present in the patient [59].

The pairwise distances between the novel subgenotypes J4 and J1 identified in this study were slightly shorter than those identified in previous genotype classifications [21]. Therefore, the identified genotypes J4 may also be interpreted as subtype- or subgenotype-level classifications. Subgenotype-level classification of SFTSV is not widely performed. Lowering the pairwise distance threshold may increase the risk of overclassification of genotypes. However, it also enabled clearer identification of regional clustering patterns. From a public health perspective, such resolution may be useful for inferring probable locations of infection and for understanding the spatial spread of SFTSV.

The regional characteristics of the SFTSV genome have been confirmed within a very localized range. However, attempting to confirm regional characteristics across wider area may render their recognition more challenging. In the future, more detailed genotype classification may clarify the relationship between genotype and regional characteristics.

We observed an association between the distribution of SFTSV genotypes and the presumed regions of infection of patients. The Miyazaki Prefecture has an area of approximately 7734.16 km^2^. The identification of multiple genotypes within this relatively limited area may be consistent with a restricted spatial spread of SFTSV; however, this interpretation remains speculative. This result is also supported by the SFTSV transmission characteristics, which, unlike highly contagious viruses such as SARS-CoV-2, is primarily transmitted through vectors (e.g., ticks) and wild animals. The home ranges of wild boars and deer, which transmit ticks, are small [60, 61]; this may have contributed to the partial geographic isolation and observed regionality of SFTSV genotypes [62].

In this study, potential reassortment and recombination signals were detected in human SFTSV strains in Japan. The results of this study suggest that genetic reassortment and recombination may have occurred between viral strains from geographically distant regions in the Miyazaki Prefecture. Furthermore, considering this along with the observed regionality of genotypes, we hypothesize that SFTSV, while generally spatially restricted, may be dispersed over long distances by birds or humans. Numerous studies have reported the role of birds in the dispersal of ticks and tick-borne pathogens; therefore, occasional long-distance dispersal of SFTSV via bird-associated tick movement may occur, although this was not directly demonstrated in the present study [18, 63, 64]. To more accurately elucidate the transmission mechanisms of SFTSV, it is necessary to investigate human-derived viral genomes as well as those from ticks and animal hosts. However, caution is warranted when interpreting recombination. The data in this study do not allow definitive confirmation that recombination has occurred in the identified strains, as sequences resembling recombination may arise from independently evolving strains in different locations.

Although no association was found between the genotypes and patient outcomes for the Miyazaki strain, the strong genetic regionality of SFTSV may have important epidemiological implications for human infection, as local viral mutations could be linked with disease severity [65]. The relationship between genotype and disease severity is expected to be clarified through more comprehensive and larger-scale analyses in the future.

This study has some limitations. First, the dataset was based solely on human-derived samples from a single geographic region, and did not include viral genomes from ticks or animal hosts, which limits direct inference of ecological and transmission mechanisms. In addition, the presumed regions of infection in this study were determined based on interviews conducted by physicians with patients or their families. Physicians identified the most likely infection regions based on the patient’s activity history and the presumed date of infection. Furthermore, in some cases, we confirmed a high likelihood of infection by visiting the presumed regions of infection and collecting ticks. However, potential misclassification of the infection regions may have affected the regional analysis in this study.

Second, as consensus sequences were constructed from amplicon-based data, PCR amplification bias and chimeric artifacts may have been introduced. The use of consensus sequences may hinder intrahost diversity detection, and these factors may produce patterns resembling recombination signals.

Third, the sample size was relatively small, which may have limited the statistical power of the analyses. Consequently, non-significant findings, including the lack of association between genotype and patient outcomes, should be interpreted with caution.

Finally, we used the classification method proposed by Yoshikawa et al. [23] for constructing the phylogenetic tree, as it accounts for the regionality of SFTSV in Japan. However, partial genome sequences were also included when this classification method was created; when they were excluded, a genotype (C5) was found wherein only one strain could be identified, depending on the segment. In this study, the genotype classification for these strains was not changed; however, we anticipate that further genomic analysis of SFTSV strains from regions outside the Miyazaki Prefecture will reveal more details on the genotypes within the Japanese clade.

## Conclusions

In this study, we demonstrated the regionality of SFTSV genotypes in the Miyazaki Prefecture, suggesting that SFTSV genotypes may have evolved under the influence of environmental factors in different regions. Furthermore, potential gene reassortment and recombination observed in this study allow us to hypothesize that, although SFTSV movement is generally limited, it may have been spread by birds and humans. The results suggest that SFTSV genome analysis may be useful for identifying presumed regions of infection in patients. Conversely, the number of patients with SFTS was limited. In the future, analysis of SFTSV genes in animals—particularly companion animals—and ticks is expected to provide a more comprehensive understanding of the regional distribution of SFTSV genotypes. Herein, we only analyzed the SFTSV genome from Miyazaki Prefecture; however, many strains from other prefectures were classified as J1 and J-unclassified, suggesting that analyzing strains from other prefectures may reveal new phylogenetic branches and improve SFTS prevention.

## Supplementary Information

Additional file 1. Number of samples used for whole genome sequencing and epidemiological analysis. Flowchart illustrating the number of samples used for whole genome sequencing and epidemiological analysis. Orange and red arrows indicate sample removal and addition, respectively.

Additional file 2. Sequences obtained in this study and reference sequences. Summary of genotypes identified after phylogenetic tree analysis. Genotypes were tabulated according to IQ-Tree results.

Additional file 3. Miyazaki, Japan, and Global trees of S, M, and L segments by the neighbor joining method. Miyazaki, Japanese, and Global trees are shown. Phylogenetic trees were constructed using the neighbor joining (NJ) method with 1000 bootstrap replicates under the Kimura 2-parameter model in MEGA (v11.0.8) [41]. Tree visualization was performed using FigTree (v1.4.4). With the neighbor joining method, no clear branching was confirmed in the Global tree for the C5 strain of the S segment; thus, it was defined as C4. The red dots on the Global tree represent the branching points of Japanese strains in J3.

Additional file 4.Enlarged view of Japanese clade. Enlarged view of the Japanese clade in the phylogenetic trees for each segment. Red and blue brackets indicate Putative J-1 and J-2, respectively.

Additional file 5. Results of RDP 4 and manual verification. Results of RDP 4 and manual verification. **a** Result of RDP 4 for LC876440. **b** result of RDP 4 for AB985298. **c** result of manual verification for LC876440 (red: potential recombinant, blue: potential minor parent, and green: potential major parent). **d** result of manual verification for AB985298 (red: potential recombinant, blue: potential minor parent, and green: potential major parent).

## Data Availability

Data supporting the main conclusions of this study are included in the manuscript.. All sequences generated in this study have been deposited in GenBank and are available under the accession numbers listed in Additional file 2.

## Abbreviations

AIC: Akaike’s information criterion
BH: Benjamini–Hochberg
IQR: Interquartile range
L segment: Large segment
M: Medium segment
ML: Maximum likelihood
NJ: Neighbor joining
NP: Nucleoprotein
PCR: Polymerase chain reaction
RT-PCR: Reverse transcription polymerase chain reaction
SFTS: Severe fever with thrombocytopenia syndrome
SFTSV: Severe fever with thrombocytopenia syndrome virus
S segment: Small segment
